# Comparative effects of 9-month in-season resistance training with a novel periodization approach (integral periodization) vs. a traditional approach on high-intensity actions and non-contact injuries in young, trained soccer players

**DOI:** 10.5114/biolsport.2025.151649

**Published:** 2025-06-06

**Authors:** Jose Jimenez-Iglesias, Oliver Gonzalo-Skok, Mario Landi-Fernández, Alejandro Perez-Bey, Eduardo de la Pascua-Roca, Pedro Gómez Piqueras, Miguel Angel Campos-Vazquez, Jose Castro-Piñero

**Affiliations:** 1 GALENO research group, Department of Physical Education, Faculty of Education Sciences, University of Cadiz, Puerto Real, Spain; 2 Sport Science Department Cádiz C.F., Cádiz C.F., Cádiz, Spain; 3 Universidad Loyola Andalucía, Department of Communication and Education, Sevilla, Spain; 4 Instituto de Investigación e Innovación Biomédica de Cádiz (INiBICA), Cádiz, Spain; 5 Institut Nacional d´Educació Física de Catalunya (INEFC), University of Lleida, Spain; 6 Paris Saint Germain Soccer Club, Paris, France; 7 Faculty of Sport, Pablo de Olavide University, Seville, Spain

**Keywords:** Strength training, Team sports, Conditioning, High-intensity actions, Periodization

## Abstract

The aim of the present study was to examine the effect of a novel periodization model – integral periodization (IP) – that considers the load balance between game-specific demands and resistance training sessions and to compare it with the effect a traditional periodization model (TP) of resistance training (RT) on fitness through lower-body muscular strength, acceleration and speed, change of direction ability, and cardiorespiratory fitness performance, in addition to non-contact injury incidence in young, trained soccer players during a full season. Forty-five (n = 21 vs n = 24) trained soccer players (15.2 ± 0.1 years) were randomly divided into a TP training group (TPG) and an IP training group (IPG). High-intensity actions (HIAs) were evaluated through countermovement jump (CMJ), squat and hip-thrust progressive loading tests, a 10-m and 30-m sprint test, a V-cut test, and a 30–15 intermittent fitness test, and non-contact injuries were recorded. In RT sessions, TPG used exercises without variability that were repeated systematically, while IPG used variable exercises for compensating the load in reference to what was experienced on the pitch. The results showed significant improvements in all tests (ES: 0.42–4.43, all p < 0.05), except the 30–15 ITF (ES: 0.21–0.27, p = 0.114–0.332), in both groups. However, IPG showed significantly better results than TPG in 10-m (p < 0.001), 30-m sprint (p < 0.001), V-cut (p < 0.012), and non-contact injuries incidence (p < 0.028). In summary, IPG showed greater HIA improvements compared to TP and reduced non-contact injury incidence in young, trained soccer players.

## INTRODUCTION

Soccer is a sport that requires the ability to perform numerous highintensity actions (HIAs), such as changes of direction (COD), decelerations, accelerations, and sprints, which are necessary for optimal performance [1]. In a soccer match, the player is exposed to a wide spectrum of HIA, including high-speed running and maximum sprints [2], changes of direction, accelerations, and decelerations [3, 4]. These HIAs seem to be decisive in sporting success, being crucial in very significant events, such as scoring, goal avoidance, assisting, and critical defensive scenarios [5]. Additionally, soccer is turning into an activity in which these actions are becoming more frequent and intense [6]. Therefore, HIA optimization should be considered in soccer players’ training.

To improve HIA, different training protocols have been proposed. While they have been discussed in the literature [7–9], it is known that game-related drills (GRD) and resistance training (RT) are fundamental to achieving this goal [10]. It has been established that periodization and consequent load management are crucial for improving HIA performance as well as decreasing the non-contact injury rate [11]. Despite this knowledge, according to the literature, the most accepted periodization models in soccer do not propose a structure that directly relates GRD and RT [12–14]. Studies have shown how to manage external load variables directly related to injury incidence, highlighting the control of chronic load in periodization and load variability between microcycles as determining factors [15]. Although much progress has been made in the study of load management in relation to GRD [12], scarce information related to RT is available [10]. There are numerous proposals that seek to improve HIA through RT; however, they do not consider the dynamics followed by players in the GRD and its potential clinical and performance impact [10]. Consequently, a deficit is identified in the load compensation through the stimulation of the structures involved during GRD and those proposed in RT sessions, which can lead to decreased performance and overuse injuries by applying repetitive forces on a tissue by reiterating common movement patterns [16]. Thus, it would be desirable to develop periodization models that would solve this deficit. Some popular RT models used in soccer [17–19] do not establish the contents of their training programmes according to the external load exerted by soccer players in the GRD. To solve this problem, IP proposes a model in which the GRD are designed in harmony with RT via a detailed analysis of the game-specific locomotor demands to generate RT sessions that complement the overloaded structures through complementary movements not overloaded in the pitch.

To our knowledge, there is no other periodization model in team sports [17–20], particularly in soccer, that designs RT programmes based on game-specific demands, avoiding iatrogenic training. On the other hand, IP proposes a model that exhaustively analyses the mechanical demands of GRD, identified through movements that are associated with HIA; based on this analysis, it establishes compensatory content at the gym with complementary movements that do not generate an overuse of structures. This novel approach makes IP a unique periodization model in soccer. For this reason, it would be interesting to compare the effects on HIA of an RT designed independently of external load and another one planned based on it. Furthermore, recent research [21] has emphasized the significance of standardizing external load quantification across various training modalities, which emphasizes the necessity of a structured approach when implementing an integrated periodization model over an extended period of time, like the nine-month intervention in the current study. Therefore, the aim of the present study was to examine the effects of a novel periodization model (IP) that considers the load relationship between game-specific demands and resistance training sessions and to compare it with a traditional periodization model (TP) of resistance training (RT) on fitness through lower-body muscular strength, acceleration and speed, change of direction (COD) ability, and cardiorespiratory fitness (CRF) performance, in addition to non-contact injury incidence in young, trained soccer players during a full season.

## MATERIALS AND METHODS

### Participants

A total of 45 U-16 soccer players (age = 15.2 ± 0.1 years) from a Professional Club Academy (LaLiga EASports, Spanish 1^st^ division) participated in the study. Players had at least 4 years of soccerspecific training experience but had not previously participated in RT programmes, and those players who did not have at least 4 years of specific soccer training were excluded from the sample. All participants completed a total of 45 microcycles (i.e., weeks) during the season, trained an average of 4 days (75 minutes per session) and one competitive match (90 minutes), as well as two weekly RT sessions (30 minutes), amounting to 450 minutes of activity per week. During the intervention, participants competed at different regionalautonomic levels in their respective categories.

All participants and their legal guardians were informed in detail of the characteristics and objectives of the study, and written consent was obtained from the parents or legal guardians. The study conformed to the Declaration of Helsinki for research involving human subjects (Declaration of Helsinki II) and was approved by the local ethics committee (University of Zaragoza – blinded for peer review).

### Experimental design

An original two-group quasi-experimental design was used. Players from two different teams were divided into a TP training group and an IP training group on a randomized basis. There were no significant between-group differences at pre- and post-intervention in anthropometric variables and biological age (Supplementary Material 1).

Data collection took place during the 2022/2023 season, and the testing weeks coincided with week 3 (pre), after 2 weeks of adaptation to the training load after the off-season period, and week 45 (post) of the season. As in other studies [22], all subjects were tested by the same raters to guarantee identical testing settings. Temperature (16.5 ± 1.76 °C) and humidity (57 ± 1.41%) were monitored during the test and retest sessions. All assessments were performed at the same location and under similar environmental conditions to minimize potential variations due to temperature and humidity. The sample of participants was larger in the first evaluation (TPG, n = 24, IPG, n = 26), but, due to clinical and club continuation reasons, some players were excluded from the final sample (TPG, n = 21, and IPG, n = 24). Anthropometric data were collected (height and body mass (BM)), and all participants undertook a field-based fitness test battery to assess HIA performance: lower body muscular strength tests through a countermovement jump test (CMJ) and squat (SQ) and hip-thrust (HT) progressive loading tests, acceleration and speed tests (10-m and 30-m sprint tests), a COD ability test (i.e., V-cut test), and a CRF (i.e., 30–15 IFT). The number and duration of non-contact injuries were also collected, as well as the total days of sick leave of the players.

To avoid negative effects of fatigue on test performance, the fieldbased fitness tests were performed on two different days, separated by a 24-hour period. On the first day, a CMJ test and a progressive loading test in SQ and HT exercises were developed, in that order. After the first day of testing, participants did not perform any extra activities. The second day, anthropometric measurements, 10-m and 30-m tests, the V-cut test, and the 30–15 IFT were assessed. The tests took place on match day (MD)-4 and MD-3 after 72 and 96 h of recovery, respectively, from the match, ensuring a dynamic recovery (low load) followed by a day of passive recovery after the match. In order to promote recovery between the two test days, at the end of the evaluations, participants performed active myofascial release work, physiotherapy treatment and specific nutritional strategies. After finishing these activities, they performed a passive rest at home, being instructed not to perform any specific activity other than rest. Thus, prior to the tests, participants had a recovery session on MD+1 of the microcycle 24 h after the match (i.e., low-load soccer tasks, nutritional strategies, physiotherapy treatment, and cryotherapy), followed by 24 h of passive recovery on MD+2. Prior to the tests, participants maintained their usual nutrition and hydration habits. Two weeks before the tests, four familiarization sessions were developed based on the execution of the tests at low-medium intensity (except for IFT 30–15) in the warm-up routine twice a week.

### Procedures

In addition to the non-contact injuries control record, the developed test followed the indications of the field-based fitness battery proposed by Jimenez-Iglesias et al. [23] (Supplementary Material 2).

### Non-contact injuries control

The club’s medical staff recorded all the injuries sustained by players throughout the intervention as well as the duration of each injury. Following the example of other studies, a player was considered fully recovered when he had full participation in training and matches [24]. For our analysis, only non-contact injuries were recorded [25]. Injuries were classified, according to severity, as “minor” (1 to 7 days), “moderately serious” (8 to 21 days), and “serious” (> 21 days) [26].

### Training protocol

All participants followed a specific soccer training programme with 4 training sessions (75–90 min) following the basis of IP as described by the authors in the work that underlies this training proposal [10]. The programme consisted of 6-week work cycles (7 cycles were completed during the intervention) divided into 2-week blocks. In each of the blocks, the load was accentuated in certain variables of fitness through a predominant use of GRD that allowed the achievement of this goal (Table 1). Despite the load accentuation, the model maintained a load stability throughout the competitive period, avoiding aggressive increases and spike loads between microcycles. Notwithstanding this stability, volume and intensity showed an inverse relationship from the beginning of the season (high volume, moderate intensity) to the end of the season (moderate volume, high-intensity) following the basic principles of traditional periodization [27].

**TABLE 1:** Main characteristics of football specific training cycle according to integral periodization. Adapted from Jiménez-Iglesias and Campos (2024).

Game-Related Drills (GRD) formats	W 1.	W 2.	W 3.	W 4.	W 5.	W 6.

	*GRD presence in the cycle*					
**Large-GRD in Medium Areas** Groups*: 8 × 8–10 × 10 RA:100–200 m^2^ LF: pace (neutral)	50%	50%	15%	15%	35%	35 %

**Medium-GRD in Big Areas** Groups*: 5 × 5–7 × 7 RA:150–250 m^2^ LF: HSR and Sprint	35%	35%	50%	50%	15%	15 %

**Small-GRD in Small Areas** Groups*: 3 × 3–4 × 4 RA:40–80 m^2^ LF: acc and dec	15%	15%	35%	35%	50%	50 %

Acc: Accelerations; Dec: Decelerations; HSR: High Sprint running; LF: Load Focus; RA: Relative Area; W: Week.

* Excluding goalkeepers.

### Resistance training programme

**TP Programme:** The TPG followed an RT programme identical to that detailed in another study carried out in a population with the same characteristics [19]. The present training programme consisted of 30–45 minutes sessions twice a week, which included a set of exercises (squats, countermovement jumps with load, hurdle jumps, jump to box, sled towing, step phase triple jumps, acceleration with COD sprint, and linear sprints) without any variation of these exercises during the season. The evolution of the load followed a linear way, progressively increasing the number of sets, repetitions, and load in 6-week blocks. This period of load was followed by a seventh week of evaluations to re-programme the loads in the squat and the CMJ exercise. These dynamics were repeated cyclically during the 9 months of the intervention. The sessions did not consider the specific soccer training programming on the pitch but followed the established periodization regardless of the dynamics and load set in the GRD proposed by the coach.

**IP Programme:** The participants followed the guidelines of the RT programme for IP described in a recent study [10]. It consisted of 2 weekly sessions on MD-4 and MD-3, with the main objective of using the training stimuli received by the structures in a balanced way to avoid overloads and minimize the risk of injury due to overuse or fatigue of the demands requested in the field. In such a manner, we adapt the neuromuscular stimuli (i.e., strength) to stimulate structures with low demands or to minimize the demand of the most requested structures during the field sessions. In the model, there are three fundamental types of GRD, each of which is characterized by a different game density that will result in the stimulation of different metrics in the soccer player: 1) Large-GRD in Medium Areas (LMA) (Groups*: 8 × 8–10 × 10 in RA of 100–200 m^2^) [28]; 2) Medium-GRD in Big Areas (MBA) (Groups*: 5 × 5–7 × 7 in RA of 150–250 m^2^) [29], characterized by a predominance of covered sprint distances and very high intensities [30]; and 3) Small-GRD in Small Areas (SSA) (Groups*: 3 × 3–4 × 4 RA of 40–80 m^2^), characterized by the presence of a large number of accelerations and decelerations [31]. The pitch soccer-specific work follows a 6-week dynamic with accentuated loads, maintaining stability within the three types of GRD, but with a greater prominence of each of them depending on the cycle we are in.

Knowing the accentuation of the load as a function of the dominant GRD, it is also possible to intuit the mechanical stress suffered by the players as a function of the overloaded metric. Therefore, the RT sessions always targeted mechanical stress compensation of the GRD (Table 2), as follows:

1. The increased presence of LMA gives rise to a neutral load, which allows the introduction of all types of movements in RT.
2. The dominance of MBAs results in a greater presence of sprints, which will overload the posterior chain, so RT sessions will use COD accelerations from low speeds and decelerations.
3. With a greater prominence of the SSA and, therefore, a greater overload on the gluteus, quadriceps, and soleus by the greater presence of accelerations from low speeds and decelerations, RT sessions will present complementary movements, such as accelerations from high speeds (hamstring approach) and changes of direction of low angulations.

**TABLE 2:** Compensatory features between GRD and resistance training sessions (Campos & Jiménez-Iglesias 2024).

	W1.	W2.	W3.	W4.	W5.	W6.
**Predominant GRD**	**LGRD-MA**		**MGRD-LA**		**SGRD-SA**	

	**PM:** without priority movements **LF:** balanced load		**PM:** accelerations with high pelvis (rolling accelerations) and top speed mechanics **LF:** hamstrings		**PM:** accelerations with low pelvis (explosive accelerations) and decelerations **LF:** gluteus, quadriceps and calf	

**RTS**	**S1:** high-speed acceleration (MD-4) **TE:** accelerations with high pelvis (rolling accelerations) and top speed mechanics **LF:** hamstrings		**S1:** mixed-multidirectional (MD-4) **TE:** changes of direction of different angles **LF:** balanced load		**S1:** high-speed acceleration (MD-4) **TE:** accelerations with high pelvis (rolling accelerations) and top speed mechanics **LF:** hamstrings	

	**S2:** accelerative-decelerative (MD-3) **TE:** accelerations with low pelvis (explosive accelerations) and decelerations **LF:** gluteus, quadriceps and calf		**S2:** accelerative-decelerative (MD-3) **TE:** accelerations with low pelvis (explosive accelerations) and decelerations **LF:** gluteus, quadriceps and calf		**S2:** mixed-multidirectional (MD-3) **TE:** changes of direction of different angles **LF:** balanced load	

Note: GRD: Game-Related Drills; LA: Large Areas; LF: Load Focus; LGRD: Large Game-Related Drills; MA: Medium Areas; MGRD: Medium Game-Related Drills; RTS: Resistance-Training Sessions; SA: Small Areas; SGRD: Small Game-Related Drills; S1: Session 1; S2: Session 2; TE: Type of Exercises; PM: Priority Movement; W: Week.

The main features of the RT programme are also shown in Table 3.

**TABLE 3:** Resistance training program features.

	exercises	series	repetitions	rest	intensity
**RTS**	Two basic exercises (Squat and Hip-Thrust with optimal power individualized load*) and 3–4 more exercises based on the movements required according to the specific football training cycle (high variability in exercises between sessions) executed in an explosive way.	2–3	4–10	3 min between sets	Moderate-light level of effort

**Increase of intensity**	Loads were increased between sessions if the player performed with good technique and explosiveness. The loads of the basic exercises (Squat and Hip-Thrust) were reprogrammed every 8 weeks. Complexity of the exercises (unilateral dominance, perturbations, cognitive distractions) increased progressively during the season.				

Note: RTS: Resistance-Training Sessions

* Velocity bar of 1 m/s

### Statistical analysis

The sample size (n = 45) was determined based on a priori power analysis, considering a moderate effect size (ES = 0.75). This effect size was chosen based on previous literature in similar studies [32] and represents a meaningful difference in context. The calculation was performed according to G*Power (version 3.1.9.6) assuming an alpha level of 0.05 and a statistical power of 80% (1-β = 0.80), ensuring sufficient sensitivity to detect moderate effects. Data distribution was assessed for normality through visual inspection of the histograms. This method was chosen to evaluate the overall shape and symmetry of the data, as it provides an intuitive understanding of distributional characteristics that might not be captured by numerical tests alone. The decision to proceed with parametric analyses was based on the visual confirmation that the data approximated a normal distribution. Descriptive data are presented as mean ± standard deviation (SD) or as percentages, as indicated in each table or figure. Paired t-tests were conducted to assess the effect of the ‘integral periodization’ intervention on the total sample. The effect size (Cohen’s d) was calculated using the pooled pre-intervention SD. The thresholds for interpreting the magnitude of Cohen’s d were set as follows: > 0.2 (small), > 0.6 (moderate), and > 1.2 (large) [33]. The percentage of change (%D) in performance variables between the two intervention groups was compared. To evaluate the relative variation of the measures before and after the intervention, the percentage change was calculated using the formula (POST-PRE)/PRE)*100; where PRE represents the initial value of the measured variables before the intervention, and POST corresponds to the value obtained after the intervention. This calculation makes it possible to determine the magnitude of the relative change in relation to the initial measurement.

To compare these changes between groups, ANCOVA was conducted with %Δ as the dependent variable, the intervention group as the fixed factor, and the pre-intervention value of the dependent variable as a covariate. The pre-intervention value was selected as a covariate to control for baseline differences and to improve the precision of the estimated intervention effects. No additional covariates were included, as no other baseline variables showed significant associations with the dependent variable that would justify their inclusion in the model. However, exploratory analyses were conducted to assess the potential influence of additional covariates, but they did not significantly alter the outcomes. This approach was used to control for baseline between-group differences. Subsequently, withingroup performance changes were analysed to determine their significance using paired t-tests, comparing post-intervention values to pre-intervention values within each group. To analyse the difference in injury frequency between the intervention groups, a chi-square test was performed with injury frequency as the dependent variable and the intervention group as the independent factor. This test was chosen to assess whether the distribution of injuries differed significantly between the ‘traditional periodization’ and ‘integral periodization’ groups. Additionally, a t-test was conducted to examine differences in injury severity.

The number of days lost due to injury was used as the dependent variable, and the intervention group served as the independent factor. This analysis was restricted to those players who sustained an injury during the study period. Statistical analyses were performed using IBM SPSS Statistics version 25 (IBM Corp., Armonk, NY, USA). The significance level for between-group and within-group comparisons was set at 0.05.

## RESULTS

### Within-group analyses

Results from within-group analyses are shown in Table 4. The TPG showed significant pre-post differences in CMJ (2.85 ± 1.37 cm, p = 0.002, ES = 0.89, CI: 1.19; 4.5), SQ (25.62 ± 3.59 kg, p = 0.000, ES = 2.93, CI: 21.28; 29.96), HT (40.95 ± 5.15 kg, p = 0.000, ES = 4.43, CI: 34.72; 47.18), and V-cut (-0.15 ± 0.1 s, p = 0.014, ES = 0.6, CI: 0.03; 0.26). In contrast, no significant pre-post differences were found in 10-m (-0.02 ± 0.03 s, p > 0.1), 30-m (-0.02 ± 0.05 s, p > 0.1), and 30–15 IFT (0.36 ± 0.37 km/h, p > 0.1).

**TABLE 4:** Changes in performance after the interval in traditional periodization group and integral periodization group (mean ± sd).

	Variable	Pre-test	Post-test	Difference (95%CI)	%Change	p-value	D-cohen^a^	
TPG (n = 21)	CMJ (cm)	35.66 ± 3.19	38.51 ± 4.88	2.85 (1.19; 4.5)	8.02 ± 9.59	0.002	0.89	Moderate

	10-m (s)	1.83 ± 0.09	1.81 ± 0.12	0.02 (-0.02; 0.06)	-0.94 ± 4.59	0.35	0.22	Small

	30-m (s)	4.41 ± 0.19	4.39 ± 0.2	0.02 (-0.04; 0.07)	-0.34 ± 2.78	0.552	0.11	Trivial

	V-cut (s)	7.05 ± 0.25	6.9 ± 0.2	0.15 (0.03; 0.26)	-1.99 ± 3.33	0.014	0.6	Small

	30–15 IFT (km/h)	19.55 ± 1.31	19.9 ± 1.29	0.36 (-0.09; 0.81)	1.97 ± 5.19	0.114	0.27	Small

	SQ (kg)	49.05 ± 8.75	74.67 ± 11.49	25.62 (21.28; 29.96)	54.68 ± 24.37	0.000	2.93	Large

	HT (kg)	41.43 ± 9.24	82.38 ± 13.51	40.95 (34.72; 47.18)	107.46 ± 53.89	0.000	4.43	Large

IPG (n = 24)	CMJ (cm)	36.97 ± 6.68	39.75 ± 4.6	2.78 (0.71; 4.85)	8.82 ± 9.68	0.011	0.42	Small

	10-m (s)	1.88 ± 0.09	1.73 ± 0.11	0.14 (0.11; 0.18)	-7.69 ± 4.57	0.000	1.56	Large

	30-m (s)	4.44 ± 0.2	4.25 ± 0.19	0.19 (0.15; 0.24)	-4.37 ± 2.51	0.000	0.95	Moderate

	V-Cut (s)	7.11 ± 0.33	6.79 ± 0.2	0.32 (0.21; 0.44)	-4.43 ± 3.61	0.000	0.97	Moderate

	30–15 IFT (km/h)	19.37 ± 1.11	19.15 ± 1.16	0.23 (-0.25; 0.71)	-1.02 ± 5.96	0.332	0.21	Small

	SQ (kg)	50.89 ± 9.26	78.1 ± 11.3	27.21 (22.87; 31.55)	56.55 ± 25.8	0.000	2.94	Large

	HT (kg)	42.45 ± 10.77	81.18 ± 10.48	38.73 (32.47; 44.99)	100.43 ± 47.25	0.000	3.6	Large

Note: CMJ: countermovement jump; HT: hip thrust; IPG: Integral Periodization Group; SQ: squat; TPG: Traditional Periodization Group; V-Cut: change of direction test; 10-m: sprint test for 10 m; 30-m: sprint test for 30 m; 30–15 IFT: 30–15 intermittent fitness test Note: For clarity, all differences are presented as improvements (positive), so that negative and positive differences are in the same direction.

The values are expressed as a percentage (%D = (POST-PRE)/PRE)*100)

P-value ≤ 0.05 denote differences statistically significant

The IPG showed significant pre-post differences in CMJ (2.78 ± 1.71 cm, p = 0.011, ES = 0.42, CI: 0.71; 4.85), SQ (27.21 ± 3.59 kg, p = 0.000, ES = 2.94, CI: 22.87; 31.55), and HT (38.73 ± 5.1 9 kg, p = 0.000, ES = 3.6, CI: 32.47; 44.99), 10-m (-0.14 ± 0.03 s, p = 0.000, ES = 1.56, CI:0.11; 0.18), 30-m (-0.19 ± 0.15 s, p = 0.000, ES = 0.95, CI: 0.15; 0.24), and V-Cut (-0.32 ± 0.09 s, p = 0.000, ES = 0.97, CI:0.21; 0.44). However, no significant pre-post differences were found in 30–15 IFT (-0.23 ± 0.39 km/h, p > 0.1).

### Between-group analyses

Groups did not show significant differences in their initial performance values in all assessed tests (Supplementary Material 3).

**FIG. 1:**
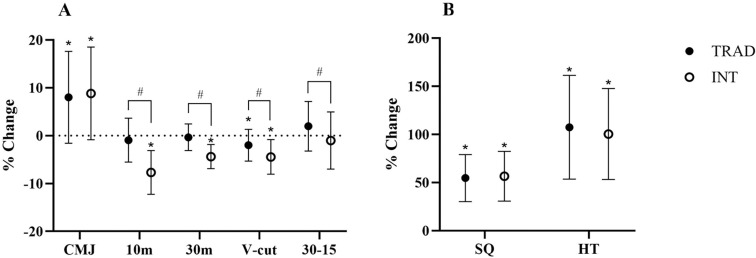
Percentage of change pre-post compared traditional periodization and integral periodization. Note: CMJ: countermovement jump; HT: hip thrust; IPG: Integral Periodization Group; SQ: squat; TPG: Traditional Periodization Group; V-Cut: change of direction test; 10-m: sprint test for 10 m; 30-m: sprint test for 30 m; 30–15 IFT: 30–15 intermittent fitness test; *Statistically significant intragroup. #Statistically significant intergroup.

Results from the between-group analyses on field-based fitness test performance are illustrated in Figure 1. All data collected pertain to those players who completed the entire intervention. Five players were excluded from the final sample (TPG, n = 24, IPG, n = 26 vs TPG, n = 21, and IPG, n = 24), and three of them were eliminated for leaving the club during the intervention and two of them for suffering long-term injuries that prevented them from continuing the intervention. Significant differences were observed between TPG and IPG in their effect on 10-m (p = 0.000), 30-m (p = 0.000), and Vcut (p = 0.012) in favour of IPG, and 30–15 IFT (p = 0.033) in favour of TPG. In contrast, no significant between-group differences were observed for CMJ (p > 0.1), SQ (p > 0.1) and HT (p > 0.1).

The results of the comparative analyses between groups regarding the incidence and severity of injuries are displayed in Table 5. Significant differences were found in injury incidence (p = 0.028) in favour of IPG, but not in the severity of them (p > 0.1).

**TABLE 5:** Incidence and severity of injuries comparing traditional periodization group and integral periodization group.

	Total players	Injuries	p-value	Severity of injury (days)	95%CI	p-value
TPG	21	12		23.33 ± 6.89	(18.95; 27.71)	

			0.028*			0.523#

IPG	24	6		21 ± 7.67	(12.95; 29.05)	

Note: IPG: Integral Periodization Group; TPG: Traditional Periodization Group. P-value ≤ 0.05 denote differences statistically significant.

* Values obtained from chi-square analysis. #Values obtained from t-test.

## DISCUSSION

The main aim of the present study was to analyse the training effect of 45 weeks (full season) of a novel RT periodization model (integral periodization) in comparison to a traditional one on HIA and noncontact injury incidence in young male trained soccer players. To the best of our knowledge, IP is the first periodization model that respects the mechanical relationships that occur in GRD and RT by establishing a detailed analysis of the movement’s patterns used in training. The main finding of this study was that, after 45 weeks of training, the lower-body muscular strength and COD ability (V-cut test) were significantly improved in both groups, while acceleration and speed only improved in the novel training periodization (i.e., IP). On the other hand, CRF did not improve in any group. Between-group comparison showed significantly better results in acceleration and speed and COD ability in the IPG with respect to the TPG. Moreover, the IPG had a significantly lower non-contact injury incidence.

In general terms, both groups showed improved performance in most tests, as demonstrated in previous studies following extended training periods [17, 34]. Improvements in the CMJ were observed in both the TPG (D: 8.02%) and the IPG (D: 8.82%), with moderate and small ESs, respectively, and with no significant betweengroup differences. Similar results were found in other studies in which young, trained soccer players performed a similar RT programme (D: 7.2%, ES: 0.58) [24]. Pertaining to SQ, we found comparable improvements in both groups (TPG: D: 54.6%, IPG: D: 56.5%) without significant difference between them. Better results were found in comparison with similar studies (D: 14%, ES: 0.75) [35]. It could be explained by the previous training background (1.5 years of experience vs. no experience), which would make the initial improvements due to RT programmes more pronounced [27]. Furthermore, our sample was younger (15.18 ± 39 years) in comparison with the other (23 ± 3.2 years), being a more advantageous period to generate neuromuscular adaptations [36].

In addition to the improvements possibly attributed to hypertrophic and neural effects of being exposed to an RT programme [1], TPG improvements in the CMJ and the SQ could be explained because both exercises were included within the training programme, and such participants were able to improve their technical execution [37]. Technical execution is closely related to intermuscular coordination, which appears to be critical for jump performance [38]. Enhanced intermuscular coordination allows players to improve muscle-tendon function by optimizing the length-tension relationship and increasing the capacity for energy transmission [39]. Additionally, other studies have shown that, even with improvements in muscle force, it is intermuscular coordination that ultimately limits jump performance [40]. The reason for the improvement in SQ in IPG might be the same as in TPG, because this exercise was included in its RT programme. On the other hand, CMJ also significantly improved in the IPG, probably due to a biomechanical similarity and the direct relationship in terms of performance and transfer that this exercise has with SQ exercise when performed with light loads [41] and with a moderate velocity loss, which seems to better enhance transfer from squat to jump through improving the ability to apply large forces in short times [42].

Both groups also improved with a large ES in HT (TPG: D: 107.4%, IPG: D: 100.43%). To our knowledge, no previous studies have assessed this variable in similar samples. As in the SQ, the same effect related to technical improvement [43] would have equally influenced improvements of IPG by including this exercise in its RT programme. However, TPG did not count this exercise in its RT programme, but with 20-m acceleration sled towing. It is known that HT has a great gluteal participation and horizontal force application [44]. Likewise, performance in the first metres of acceleration depends largely on the correct gluteal activation and horizontal force application [45]. Therefore, the exercises could have influenced each other by biomechanical and neuromuscular similarities [27]. In addition, incorporating SQ into the training programme might have induced similar adaptations in the gluteus muscles and contributed to improvements in HT performance [44].

Only IPG obtained significant improvements with large and moderate ES in 10 m and 30 m, respectively (10 m, D: -7.69%, 30 m, D: -4.37%). Our results were slightly different from those obtained by other studies with similar samples and RT programmes (10 m, D: -1.25%, ES: 0.15) [24], (30 m, D: -2.4%, ES: 0.80) [46]. Asencio et al. (2024) [47] found significant but smaller improvements in a similar sample in 10 m after following an RT programme (G1, D: -2.06%, ES: 0.36; G2, D: -4.02%, ES: 0.72) and no significant improvements in 30 m (G1, D: 1.41%, ES: -0.31; G2, D: -0.24%, ES: 0.05). The authors explain their findings due to the potential effect of neuromuscular fatigue during competitive microcycles, an aspect that is alleviated with IP due to the mechanical compensation of pitch training and RT load. In our intervention, a much higher D% was obtained in both tests in comparison with the aforementioned studies, probably because we used exercises similar to the accelerative positions in a multicomponent programme. Such previous studies have used the YoYo half SQ and YoYo leg curl [24], and only the YoYo leg curl [46], two exercises with much less dynamic correspondence [48], so that the improvement could be due to tissue adaptations rather than technical and qualitative elements. Similarly to other studies [19] with similar samples and RT programmes as the TPG, no significant improvements in linear velocity (20-m sprint test) were obtained. Moreover, IPG presented significantly greater improvements than TPG in 10 m (D: -7.69% vs D: -0.94%) and 30 m (D: -4.37% vs D: -0.34%), which might be due to the fact that RT, followed by IPG, had more exercises aimed at the posterior chain, the most determinant musculature in acceleration and speed abilities [49].

In relation to COD ability, we observed significant improvements with moderate ES in both groups. The IPG showed significantly greater improvements (D: -4.43%) than the TPG (D: -1.99%). These differences may be explained by the fact that players used a variety of movement planes and vectors, considering COD performance as highly dependent on the ability to apply force in multiple planes and vectors [50]. Furthermore, IPG also used isoinertial devices, which are more effective in improving COD ability than conventional devices [51], due to the ability to stimulate power in eccentric-concentric actions. In addition, they allow an eccentric overload while performing multidirectional tasks [51]. Similarly, the exercises proposed by the IPG exhibited a ballistic nature with high dynamic correspondence, as well as exposure to dynamic stretching of musculoskeletal structures, which contributes to enhanced COD performance [52]. Other studies carried out with similar samples and RT programmes to IPG obtained slightly better improvements (COD, D: -5%, ES: 0.52) [53], probably due to a shorter duration (7 weeks vs. 45 weeks) and the period (in-season vs. at the end of the season), when fatigue elements could influence explosive performance, including COD ability [54].

Finally, we did not find significant improvements in CRF in either group. The literature lacks studies in which young, trained soccer players evaluated this test after an RT programme. However, some studies argue that CRF does not improve at the end of the season due to aspects related to fatigue and the accumulation of competitive load [55]. The high external and cognitive load to which soccer players are exposed throughout the season could induce central fatigue mechanisms that would directly influence the overall physical performance of the players by affecting the CRF [54], explaining the current results found.

In clinical settings, there were no significant between-group differences in the severity of non-contact injuries. However, there were differences in the number of them, with 50% less in the IPG despite having 3 more players in their sample. Despite having three more players in the sample, the IPG experienced a 50% decrease in noncontact injuries, which has significant clinical ramifications for soccer. A significant percentage of injuries in sports are non-contact injuries, which occur when players do not directly touch one another. These injuries can impair team performance, raise medical expenses, and restrict a player’s availability throughout the season [56]. This could be due to several reasons. Firstly, the IPG established RT sessions with a specific mechanical analysis seeking compensation for overloaded movements in the GRD, while the TPG established the RT sessions in a fixed manner without contemplating the priority dynamics in the GRD cycle. It is known that overuse of structures and mechanical stress generated by GRD are among the major risk factors for non-contact injuries in soccer players [11]. Therefore, compensating for this mechanical load without overloading it could have been a productive strategy in the IPG. On the other hand, another reason that might explain these results is that, while the TPG executed its RT exercise programme without any type of variability, IPG used constant variability in the type of exercises and in their execution. It has been reported that variable RT programmes seem to be superior to protocols without variability [57], by avoiding repeated loads with consequent degradation of structures as well as by promoting greater neuroplastic adaptations that generate more adaptive capacity to potentially injurious motor situations [58].

The main limitations of this study are the absence of a control group with which to compare the intervention groups as well as an objective external load monitoring load with a global positioning system or other devices. Another potential limitation is selection bias, as the sample consisted exclusively of players from a single academy, forming a closed sample, a common limitation in many studies carried out in the field of performance. This could restrict the findings’ applicability to larger soccer groups, such as players from various training settings or competitive levels. To minimize selection bias, all eligible players within the academy were included in the study, and no additional selection criteria were applied beyond participation in the training programme. As strengths, it was an intervention that covered a complete season in a professional soccer training context.

## CONCLUSIONS

In conclusion, a full season (45 microcycles) of IP with an RT programme seemed to have positive effects on the improvement of HIA, such as lower-body strength, acceleration and speed, and COD ability, but did not seem to improve CRF. Therefore, the results of this study suggest that the IP seems to be much superior in improving HIA and reducing the number of non-contact injuries than a TP in young, trained soccer players. These findings may have implications for managing training programmes to increase soccer players’ performance as well as to reduce their risk of injury.

## Supplementary Material

Comparative effects of 9-month in-season resistance training with a novel periodization approach (integral periodization) vs. a traditional approach on high-intensity actions and non-contact injuries in young, trained soccer players
