# Supplementary material for: Comparative effects of 9-month in-season resistance training with a novel periodization approach (integral periodization) vs. a traditional approach on high-intensity actions and non-contact injuries in young, trained soccer players
Source: Biol Sport. 2025 Jun 6;42(4):261–74. doi: 10.5114/biolsport.2025.151649 (PMC12492344; doi:10.5114/biolsport.2025.151649)
Supplement: Comparative effects of 9-month in-season resistance training with a novel periodization approach (integral periodization) vs. a traditional approach on high-intensity actions and non-contact injuries in young, trained soccer players [file JBS-42-4-56156-s1.pdf]

REFERENCES

SUPPLEMENTARY MATERIAL 3.
Comparison of baseline values between TPG and IPG (mean ± SD).

Variable	TPG	IPG	p-value
	(N = 21)	(N = 24)	
CMJ (cm)	35.66 ± 3.19	36.97 ± 6.68	0.416
10-m (s)	1.83 ± 0.09	1.88 ± 0.09	0.069
30-m (s)	4.41 ± 0.19	4.44 ± 0.2	0.529
V-Cut (s)	7.05 ± 0.25	7.11 ± 0.33	0.513
30–15 IFT (km/h)	19.5 ± 1.31	19.37 ± 1.11	0.636
SQ (kg)	49.05 ± 8.75	50.89 ± 9.26	0.49
HT (kg)	41.43 ± 9.24	42.45 ± 10.77	0.735

Note: CMJ: countermovement jump; HT: hip thrust; IPG: Integral Periodization Group; SQ: squat; TPG: Traditional Periodization Group; V-Cut: change of direction test; 10-m: sprint test for 10 m; 30-m: sprint test for 30 m; 30–15 IFT: 30–15 intermittent fitness test
P-value ≤ 0.05 denote differences statistically significant
